# Prevalence of chronic infections and susceptibility to measles and varicella-zoster virus in Latin American immigrants

**DOI:** 10.1186/s40249-016-0136-7

**Published:** 2016-05-11

**Authors:** Yves Jackson, Lilian Santos, Isabelle Arm-Vernez, Anne Mauris, Hans Wolff, François Chappuis, Laurent Getaz

**Affiliations:** Division of Primary Care Medicine, Geneva University Hospitals and Faculty of Medicine, University of Geneva, Rue Gabrielle Perret Gentil 6, Geneva, 1211 Switzerland; Institute of Global Health, University of Geneva, Geneva, Switzerland; Division of Tropical and Humanitarian Medicine, Geneva University Hospitals and Faculty of Medicine, University of Geneva, Geneva, Switzerland; Department of Genetics and Laboratory Medicine, Geneva University Hospitals and Faculty of Medicine, University of Geneva, Geneva, Switzerland; Division of Correctional Medicine and Psychiatry, Geneva University Hospitals and Faculty of Medicine, University of Geneva, Geneva, Switzerland

**Keywords:** Immigrants, Europe, Chronic infection, Co-infection, Trypanosma cruzi, Strongyloides stercoralis, HIV

## Abstract

**Background:**

Large numbers of Latin American immigrants recently arrived in Western Europe. Curative and preventive programmes need to take account of their risk of suffering and transmitting imported chronic infections and of their susceptibility to cosmopolitan infections. We aimed to assess the prevalence and co-occurrence of imported chronic infections among Latin American immigrants, and their susceptibility to highly prevalent cosmopolitan infections.

**Methods:**

Adult participants were recruited in the community and in a primary health centre in Geneva in 2008. Serological tests were performed on stored sera for HIV, HBV, syphilis, *Strongyloides stercoralis*, *Trypanosoma cruzi*, varicella and measles. We considered only chronic active infections in the analysis.

**Results and discussion:**

The 1 012 participants, aged 37.2 (SD 11.3) years, were mostly female (82.5 %) and Bolivians (48 %). Overall, 209 (20.7 %) had at least one and 27 (2.7 %) two or more chronic infections. *T. cruzi* (12.8 %) and *S. stercoralis* (8.4 %) were the most prevalent chronic active infections compared to syphilis (0.4 %), HBV (0.4 %) and HIV (1.4 %). Concomitant infections affected 28.2 and 18.5 % of *T. cruzi* and *S. stercoralis* infected cases. Bolivian origin (a*OR*: 13.6; 95 % *CI*: 3.2–57.9) was associated with risk of multiple infections. Susceptibilities for VZV and measles were 0.7 and 1.4 %, respectively. Latin American immigrants are at risk of complications and possible reactivation of chronic parasitic infections but have overall low risks of chronic viral and syphilitic active infections.

**Conclusions:**

Systematic screening for chronic active parasitic infections is therefore necessary especially among Bolivians. The high protection rate against measles and VZV doesn’t require specific preventive interventions.

**Electronic supplementary material:**

The online version of this article (doi:10.1186/s40249-016-0136-7) contains supplementary material, which is available to authorized users.

## Multilingual abstracts

Please see Additional file 1 for translations of the abstract into the five official working languages of the United Nations.

## Background

Recent decades have witnessed a sharp increase in transnational human mobility. In 2013, international migrants stock amounted to 231 million (3.2 % of the world population) with Europe hosting 72 million [1]. Latin Americans (Latinos) represent a major group of immigrants in OECD countries, accounting for approximately 25 % of the total stock and their number has grown by 36 % in the past decade. It is estimated that around 4 millions recently settled in Western Europe [2]. While the prevalence and incidence of non-communicable diseases rapidly increase in Latin America, the burden of communicable diseases remains a major public health and economic problem [3].

Gradients in prevalence and susceptibility to specific infectious diseases between immigrants and autochthonous population impact on pathogens circulation in receiving countries and challenge healthcare systems to adapt to emerging needs [4]. For instance, the emergence of Chagas disease in Europe has been shown to result from (i) the rapid influx of people from endemic countries, the majority being women of child-bearing age susceptible to vertically transmit *T. cruzi*, (ii) insufficient pre-existing health policies, (iii) lack of health professionals awareness and (iv) delayed responses of healthcare systems [5, 6].

Previous studies showed that Latinos immigrants in Europe bear a significant risk of chronic infections with a potential of reactivation and transmission, such as Chagas disease and latent tuberculosis [5, 7]. Coexisting chronic infections in immigrants has triggered interest because of the recognition of shared risk factors, possible clinical interactions, especially in presence of immunosuppression, and frequent delay in diagnosis [8, 9]. In Geneva for instance, the mean duration before *Trypanosoma cruzi* infection (Chagas disease) was diagnosed was 4.9 years, and 97.4 % patients were unaware of being infected at time of diagnosis [10]. Chronic forms of Hepatitis B [11], HIV [12], syphilis [13] and *Strongyloides stercoralis* [14] infections are more prevalent in Latin America than in Western Europe. A better understanding of the activity, distribution and overlap of these diseases within the Latino community in Europe is thus necessary to implement appropriate clinical and public health strategies.

Immigrants may also bear risks of suffering from and enhancing transmission of highly prevalent cosmopolitan infections when lacking specific immunity. Outbreaks of rubella and chickenpox that predominantly affected previously unexposed or non vaccinated immigrants in Western countries exemplify this vulnerability [15, 16]. In Switzerland, recent measles outbreaks have affected adult Latino immigrants [17]. Varicella-zoster virus (VZV) prevalence is lower in tropical countries than in Europe, therefore immigrants arriving in Europe are at risk of primary infection [18]. Adult immunization is highly effective in preventing potentially severe complications in persons at risk. So far, the lack of data has hampered decision-making about immunization programmes implementation for Latino immigrants.

This study aims at assessing the prevalence and the co-occurrence of HIV, hepatitis B, syphilis, *S. stercoralis* and *T. cruzi* (Chagas disease) infection and the susceptibility to varicella-zoster and measles virus among Latin American immigrants in the community, and to compare Bolivians to migrants from other origins.

## Methods

### Setting

Geneva Canton (population 480 000) hosts a community of recently arrived immigrants from diverse Latin American countries. Many live without residency permit or health insurance and face difficulties accessing to care. The Geneva University Hospitals (HUG) community care centre is the gateway to the public healthcare system for vulnerable immigrants. It provides preventive, curative and rehabilitation care.

### Design

This cross-sectional retrospective study used blood samples and demographic data collected during a Chagas disease epidemiological survey conducted in 2008 [10]. It received ethical clearance from the Geneva University Hospitals ethical board (protocol 07-285).

### Participants and procedures

The study was implemented in the community, with the support of Latino churches and social organizations, and at the HUG community care centre. Cultural mediators and documentation in Spanish were designed to maximize participation. Eligible participants were all consecutive adults (>16 year) Latino immigrants presenting at the health centre or attending community meetings in churches and cultural organizations between June and December 2008. All participants provided detailed informed consent that included the possible subsequent use of collected sera for other infectious diseases diagnostic studies. Pregnant women were excluded from this study and referred to the Division of Obstetrics for another study. Sociodemographic data were collected by questionnaire. Rural area was defined as living in a village or in any non-urban setting in the campaign.

### Serological testing

The analyses were performed in 2015 on serum samples stored at −20 °C in the Geneva University Hospitals’ biobank. After unfreezing, sera were tested for HIV 1 and 2 antibodies and p24 antigen (Architect HIV Ag/Ab Combo assay, Abbott Laboratories, Abbott, USA). Positive results were confirmed with VIDAS HIV Duo Quick assay (bioMérieux, Marcy, France). Hepatitis B infection was assessed first by testing for antibodies against core antigen (anti-HBcAb) (Architect Anti-HBc Ig assay, Abbott) and then for the surface antigen (HBsAg) (Architect HBsAg Qualitative II assay, Abbott) in anti-HBc positive cases. Positive HBsAg cases were reported as having chronic active Hepatitis B virus infection. The diagnostic algorithm for syphilis (*Treponema pallidum*) was based on a treponemal ELISA assay (Architect Syphilis TP, Abbott) as a first-line screening test. Positive samples were further tested with non-treponemal (RPR-nosticon II, bioMérieux) and treponemal (Cellognost-Syphilis TPHA assays, Siemens, Marburg, Germany) assays. Syphilitic infection was considered in presence of positive treponemal and non-treponemal tests. Cases of positive TPHA with negative RPR testing were not included in the final analysis, as indicative of cured or treated syphilis or rarely late latent syphilis. Serological testing for *S. stercoralis* was performed with an ELISA assay (Strongyloides ratti EIA, Bordier, Crissier, Switzerland). Details on *T. cruzi* testing performed in 2008 are provided elsewhere [10]. Susceptibility to VZV and measles virus infections was defined by negative serological testing with Anti-VZV/IgG assay (Virion Labordiagnostik, Zürich, Switzerland) and Anti-Measles Enzygnost /IgG (Siemens), respectively. Borderline and positive results were further tested with VIDAS varicella-zoster (IgG) and VIDAS Meales (IgG) (bioMérieux) confirmatory tests. Co-infection was considered in presence of more than one infection.

### Statistical analysis

Categorical data were presented as absolute numbers and proportions, and continuous variables as mean and standard deviation (SD). To investigate the relation between co-infection, respectively S. strongyloides infection, and possible predictive factors, we used 2 × 2 tables and performed chi-square and Fisher’s exact tests for categorical variables and unpaired Student’s t-tests for continuous variables. Univariate and multivariate logistic regression analysis were used to assess factors associated with co-infection respectively S. strongyloides infection. Adjustment was performed for age, sex, coming from rural vs. urban area, Bolivian origin and having left Latin America since 5 years. Data were analysed using IBM SPSS Statistics (version 23).

## Results

### Participants

Overall, 1 012 participants were included with a mean age of 37.2 (SD 11.3) years, a female predominance (83 %) and origin from 17 countries. The most frequently origins were Bolivia (*n*=486; 48 %), Brazil (*n*=249; 25 %), Colombia (*n*=61; 6 %), Peru (*n*=58; 6 %) and Ecuador (*n*=47; 5 %).

### Serological tests results

Table 1 summarizes the participants’ socio-demographic characteristics and the serological tests results stratified by origin. We found a 12.8 and 8.4 % prevalence of chronic parasitic infections with *T. cruzi* and *S. stercoralis*. Details about *T. cruzi* infection were published elsewhere [10]. Importantly, it was more frequent in Bolivians than in participants from other origins (26.1 % versus 0.6 %, *P* <0.001). After adjustment, risk factors for *S. stercoralis* infection included male gender, Bolivian origin and shorter duration of stay in Europe (Table 2).

**Table 1:** Sociodemographic characteristics, prevalence of infection and susceptibility to local infections of the total population (*n*=1012) and stratified by origin

	Total population (*n*=1012)	Bolivians (*n*=486)	Other origin (*n*=526)	*P*- value
	Mean (SD) or *n* (%)	Mean (SD) or *n* (%)	Mean (SD) or *n* (%)	
Age	37.4 (11.3)	36.5 (10.8)	38.3 (11.7)	0.01
Sex (female)	835 (82.5 %)	404 (83.1 %)	431 (81.9 %)	0.62
Coming from urban area	813 (80.3 %)	373 (76.7 %)	440 (83.7 %)	0.007
Years since arrival in Europe	4.9 (4)	4.5 (2.9)	5.3 (4.8)	0.001
Prevalence of infections				
HIV	14 (1.4 %)	3 (0.6 %)	12 (2.3 %)	0.04
HBV (anti-HBc Ab+)	75 (7.4 %)	40 (8.2 %)	35 (6.7 %)	0.40
HBV (HBsAg+)	4 (0.4 %)	2 (0.4 %)	2 (0.4 %)	1
Syphilis (any reactive test)	24 (2.4 %)	9 (1.9 %)	15 (2.9 %)	0.66
Syphilis infection	4 (0.4 %)	2 (0.4 %)	2 (0.4 %)	1
*T. cruzi*	130 (12.8 %)	127 (26.1 %)	3 (0.6 %)	<0.001
*S. stercoralis*	85 (8.4 %)	65 (13.4 %)	20 (3.8 %)	<0.001
Susceptibility to cosmopolitan infections:				
VZV susceptibility	7 (0.7 %)	1 (0.2 %)	7 (1.1 %)	0.126
Measles susceptibility	14 (1.4 %)	4 (0.8 %)	10 (1.9 %)	0.181

**Table 2:** Factors associated with *Strongyloides stercoralis* infection (*n*=1012)

Factor	*OR* (95 % *CI*)	*OR* adjusted^a^ (95 % *CI*)
Age >35	1.08 (0.69–1.69)	1.41 (0.88–2.25)
Male	1.86 (1.12–3.10)	2.06 (1.21–3.51)
Bolivian origin	3.91 (2.33–6.55)	3.83 (2.27–6.47)
Coming from a rural area	1.69 (1.03–2.79)	1.59 (0.95–2.68)
Less than 5 years out of Latin America	2.10 (1.29–3.40)	2.18 (1.32–3.62)

^a^Adjusted for all factors in table

Global HIV prevalence was 1.4 % with non-Bolivian being more frequently infected (*P* <0.05). Regarding hepatitis B, 7.4 % participants had anti-HBc antibodies while 0.4 % had a positive test for HBs antigen, indicative of active infection. Overall, 2.4 % had serologic markers of cured syphilis while 0.4 % had positive treponemal and non treponemal tests indicating active infection. Rates did not differ between Bolivians and others. Susceptibility to VZV and measles were 0.7 and 1.4 %. Measles immunity decreased with younger age. Nine out of 252 persons (3.6 %) born after 1980 were susceptible to measles, whereas five out of 760 (0.7 %) older ones were susceptible (*P* <0.001).

One or more chronic active infections were found in 209 participants (20.7 %) with a majority presenting only one infection. Twenty-seven (2.7 %) had two or more infections (Table 3). Of those, 24 were co-infected with *T. cruzi* and *S. stercoralis*, which meant 18.5 % of *T. cruzi* infected participants presented *S. strongyloides* co-infection and 28.2 % vice versa. Only two (14 %) of the 14 HIV patients presented with a co-infection.

**Table 3:** Number of chronic active infections in Latino-American immigrants (*n*=1012)

Chronic infections	Total population (*n*=1012) *n* (%)	Bolivians (*n*=486) *n* (%)	Other origin (*n*=526) *n* (%)
0	803 (79.3 %)	313 (64.4 %)	490 (93.1 %)
1	182 (18 %)	148 (30.5 %)	34 (6.5 %)
2	26 (2.6 %)	24 (4.9 %)	2 (0.4 %)
3	1 (0.1 %)	1 (0.2 %)	0 (0 %)
≥1	209 (20.7 %)	173 (35.6 %)	36 (6.8 %)

### Factors associated with infection

Tables 4 and 5 show factors associated with infection overall and with multiple infections. After adjustment, age, male gender, Bolivian and rural origin were predictors of infection. Bolivian and rural origin were significantly associated with multiple infections, but only the former remained significant after adjustment for the other factors.

**Table 4:** Factors associated with infection (*n*=209)

Factor	*OR* (95 % *CI*)	*OR* adjusted^a^ (95 % *CI*)
Age >35	1.85 (1.35–2.53)	2.50 (1.75–3.57)
Male	1.40 (0.96–2.04)	1.78 (1.17–2.73)
Bolivian origin	7.52 (5.11–11.07)	8.09 (5.44–12.03)
Rural origin	1.80 (1.27–2.57)	1.62 (1.09–2.39)
Less than 5 years out of Latin America	1.20 (0.88–1.63)	1.39 (0.98–1.97)

^a^Adjusted for all factors in table

**Table 5:** Factors associated with multiple infections (*n*=27)

Factor	*OR* (95 % *CI*)	*OR* adjusted^a^ (95 % *CI*)
Age >35	1.20 (0.56–2.59)	1.42 (0.64–3.19)
Male	1.68 (0.70–4.03)	1.91 (0.78–4.70)
Bolivian origin	14.21 (3.35–60.31)	13.59 (3.19–57.91)
Rural origin	2.48 (1.12–5.50)	2.14 (0.95–4.84)
Less than 5 years out of Latin America	1.41 (0.64–3.12)	1.42 (0.62–3.24)

^a^Adjusted for all factors in table

## Discussion

This community-based study showed that 20.7 % Latinos in Geneva suffered from one or more chronic infections with a risk of reactivation and of transmission. Infection with *T. cruzi* and *S. stercoralis* accounted for the heaviest burden in this predominantly Bolivian population, whereas HIV, HBV and syphilis were less frequent. Age, male gender, rural and Bolivian origin predicted the risk of suffering of at least one chronic infection, and Bolivians were at higher risk of multiple infections. Immigrants showed high protection rates against highly prevalent cosmopolitan infections such as VZV and measles.

Several limitations should be considered. The first pertains to the predominant Bolivian population reflecting the distribution of Latino immigrants in Geneva. In Europe, immigrants from Ecuador, Colombia, Peru, Brazil and Argentina are more numerous [2]. The high prevalence of Chagas disease in Bolivia skewed our results about co-infections rates, as *T. cruzi* accounted for the major cause of infection in our sample. Therefore, studies conducted with immigrants of other origins may entail different results. Secondly, albeit cold storage, sample handling and serological tests have been performed under optimal conditions, we cannot exclude that these conditions impacted on the reactivity of some sera stored for 6 years. Moreover, the use of a single diagnostic test for *S. stercoralis* may entail a limited risk of suboptimal discrimination capacity between recently treated and current infection [19]. Thirdly, we restricted our study to a limited number of pathogens. Prevalence of other pathogens causing chronic infection in Latino immigrants Europe, such as *Schistosoma spp*, *Leishmania spp*, *Plasmodium spp*, Hepatitis C and HTLV, have been shown to be very low and of limited clinical and public health significance [20–23]. We cannot exclude that some participants presented with other chronic infections, notably latent tuberculosis [20]. Finally, women were over-represented in our sample, whereas genders are equally represented among Latino immigrants in Europe [2]. Considering that males tended to have more risk of multiple infections (notably with *S. stercoralis*), the proportion of co-infections might be higher in settings with a more balanced distribution.

This study investigated the largest sample of Latinos immigrants in Europe so far. The community-based recruitment and the restriction to infections at the chronic stages offer new clinical and epidemiological insights. For instance, the difference between the 2.4 % overall syphilis seroreactivity in Geneva, with 0.4 % showing active infection, compared to 3.5–6.4 % in Spain, likely reflect the population-based sample as opposed to more selective samples in other studies [7, 20, 24]. In our sample, HBV prevalence was slightly lower than reported in previous studies likely for similar reasons [7, 20, 25]. Differences were less marked regarding HIV prevalence, which is consistently low among Latinos in Europe as compared to other immigrants groups [7, 20, 22, 26]. Our findings do not support recent recommendations to systematically screen asymptomatic Latino immigrants for HIV, syphilis and HBV [21]. We rather suggest tailoring screening strategies according to individual risk assessment for these pathogens.

Studies conducted in Italy and Spain with large populations of Bolivian immigrants have consistently reported comparably high prevalence of *T. cruzi* infection, confirming that this group bears the highest burden of infection among Latino immigrants [27, 28]. This supports universal screening in Bolivian immigrants with a focus on groups at higher risk of transmission or complication or higher chance of successful treatment, such as child-bearing age or pregnant women, people with immunosuppression, newborns and children.

We found an overall 8.4 % prevalence of *S. stercoralis* infection in our population, with Bolivians, from rural origin and men showing the highest rates, which correlates with community studies conducted in Latin America [29]. To the best of our knowledge, this is the first community-based survey among Latinos in Europe. Ramos et al. found 26.8 % of Latino patients of a tertiary hospital in Spain being infected, with Ecuadorians showing highest risk of infection [22]. A study among HIV-infected Latinos in Italy showed 6.7 % prevalence [30]. We found frequent parasitic co-infections between *T. cruzi* (18.5 %) and *S. stercoralis* (28.2 %) confirming previous findings from Italy [31]. In our analysis, male gender, Bolivian origin and shorter duration of stay were positively associated with infection. The lack of significant association with rural origin may pertain to a limited study power. Adding to the current practice of screening Latinos with eosinophilia for *S. stercoralis*, our findings support systematic detection of immigrants at risk, notably Bolivians, for both parasites. Moreover, screening for infection in persons with ongoing or risk of immunosuppression before initiating steroid therapy and chemotherapy is essential to prevent the occurrence of hyper infestation [30].

Our data showed low susceptibility to measles and varicella-zoster infection in Latinos. This is of epidemiological and clinical relevance considering the community circulation of these pathogens in Switzerland and that most participants were women of child-bearing age and predominantly active in the domestic work industry. The 98.6 % protection against measles is actually higher than among Geneva residents [17]. We found younger immigrants being slightly more susceptible. In absence of comparative data in Europe, we cannot ascertain this finding is generalizable to neighbouring countries. In 2004, Latino immigrants in Canada had an overall 88 % protection rate, with women more at risk of infection than men [32]. Immunization campaigns implemented in Latin America in the 1980’s sharply reducing the virus circulation and the incidence [33]. Therefore, we can safely consider that adult Latino immigrants, especially those borne before 1980 are at very low risk for measles in Switzerland and do not need systematic screening or catch-up immunization. However, as measles is no longer circulating in Latin America, unvaccinated children and young adults originating from countries characterized by suboptimal vaccination coverage must be considered susceptible. Regarding VZV, we found higher rates of protection than previously reported among immigrants from Surinam and the Antilles in the Netherlands and HIV infected immigrants in Spain [34, 35]. In Catalonia, Valerio et al. found a two-fold increase in VZV infection incidence rate among Latino adults compared to Spanish [36]. Yet, our findings call against implementation of systematic screening in this group but rather supports the current strategy of screening and immunizing immunosuppressed adults and providing post-exposure prophylaxis to pregnant women [37]. Further studies are needed to evaluate immigrants’ susceptibility to other local frequently acquired and vaccine-preventable infections such as pertussis.

## Conclusions

In summary, our findings support the need to systematically screen adult Latin American immigrants, especially Bolivians, against chronic parasitic infections and to tailor screening strategies for other infections and susceptibility to measles and VZV after individual risk assessment.

## Additional file

Additional file 1: Multilingual abstracts in the five official working languages of the United Nations. (PDF 384 kb)

## Abbreviations

AOR: adjusted odd ratio
CI: confidence interval
HBV: hepatitis B virus
HIV: human immunodeficiency virus
HUG: Geneva University Hospitals
VZV: varicella zoster virus

## References

[1] OECD-UNDESA. World migration in figures 2013. Available from: https://www.oecd.org/els/mig/World-Migration-in-Figures.pdf. Accessed 28 May 2015.

[2] IOM (2012). Rutas y dinámicas migratorias entre los países de América Latina y el Caribe (ALC), y entre ALC y la Unión Europea.

[3] GBD 2013 Mortality and Causes of Death Collaborators (2015). Global, regional, and national age-sex specific all-cause and cause-specific mortality for 240 causes of death, 1990–2013: a systematic analysis for the Global Burden of Disease Study 2013. Lancet.

[4] Sharma S, Carballo M, Feld JJ, Janssen HL (2015). Immigration and viral hepatitis. J Hepatol.

[5] Basile L, Jansa JM, Carlier Y, Salamanca DD, Angheben A, Bartoloni A (2011). Chagas disease in European countries: the challenge of a surveillance system. EuroSurveill.

[6] Requena-Mendez A, Albajar-Vinas P, Angheben A, Chiodini P, Gascon J, Munoz J (2014). Health policies to control Chagas disease transmission in European countries. PLoS Negl Trop Dis.

[7] Bocanegra C, Salvador F, Sulleiro E, Sanchez-Montalva A, Pahissa A, Molina I (2014). Screening for imported diseases in an immigrant population: experience from a teaching hospital in Barcelona, Spain. Am J Trop Med Hyg.

[8] Getaz L, Chappuis F, Lozano Becerra JC, Wolff H, Albajar-Vinas P (2014). Persistent tropical diseases among migrants. Rev Med Suisse.

[9] Hochberg NS, Moro RN, Sheth AN, Montgomery SP, Steurer F, McAuliffe IT (2011). High prevalence of persistent parasitic infections in foreign-born, HIV-infected persons in the United States. PLoS Negl Trop Dis.

[10] Jackson Y, Getaz L, Wolff H, Holst M, Mauris A, Tardin A (2010). Prevalence, clinical staging and risk for blood-borne transmission of Chagas disease among Latin American migrants in Geneva, Switzerland. PLoS Negl Trop Dis.

[11] Trepo C, Chan HL, Lok A (2014). Hepatitis B virus infection. Lancet.

[12] UNAIDS (2013). UNAIDS report on the global AIDS epidemic 2013.

[13] WHO TDR. Disease watch focus: syphilis. Accessed on 28 05 2015. Available from: http://www.who.int/tdr/publications/disease_watch/syphilis/en/.

[14] Buonfrate D, Mena MA, Angheben A, Requena-Mendez A, Munoz J, Gobbi F (2015). Prevalence of strongyloidiasis in Latin America: a systematic review of the literature. Epidemiol Infect.

[15] Getaz L, Siegrist CA, Stoll B, Humair JP, Scherrer Y, Franziskakis C (2010). Chickenpox in a Swiss prison: susceptibility, post-exposure vaccination and control measures. Scand J Infect Dis.

[16] Lemos C, Ramirez R, Ordobas M, Guibert DH, Sanz JC, Garcia L (2004). New features of rubella in Spain: the evidence of an outbreak. Euro Surveill.

[17] Delaporte E, Richard JL, Wyler Lazarevic CA, Lacour O, Girard M, Ginet C (2011). Ongoing measles outbreak, Geneva, Switzerland, January to March 2011. Euro Surveill.

[18] Kjersem H, Jepsen S (1990). Varicella among immigrants from the tropics, a health problem. Scand J Soc Med.

[19] Bisoffi Z, Buonfrate D, Segui M, Mejia R, Cimino RO, Krolewicki AJ (2014). Diagnostic accuracy of five serologic tests for Strongyloides stercoralis infection. PLoS Negl Trop Dis.

[20] Manzardo C, Trevino B, Gomez i Prat J, Cabezos J, Mongui E, Claveria I (2008). Communicable diseases in the immigrant population attended to in a tropical medicine unit: epidemiological aspects and public health issues. Travel Med Inf Dis.

[21] Monge-Maillo B, Lopez-Velez R, Norman FF, Ferrere-Gonzalez F, Martinez-Perez A, Perez-Molina JA (2015). Screening of imported infectious diseases among asymptomatic sub-Saharan African and Latin American immigrants: a public health challenge. Am J Trop Med Hyg.

[22] Ramos JM, Leon R, Andreu M, Parras ER de l, Rodriguez-Diaz JC, Esteban A (2015). Serological study of Trypanosoma cruzi, Strongyloides stercoralis, HIV, human T cell lymphotropic virus (HTLV) and syphilis infections in asymptomatic Latin-American immigrants in Spain. Trans R Soc Trop Med Hyg.

[23] Salvador F, Molina I, Sulleiro E, Burgos J, Curran A, Van den Eynde E (2013). Tropical diseases screening in immigrant patients with human immunodeficiency virus infection in Spain. Am J Trop Med Hyg.

[24] Sampedro A, Mazuelas P, Rodriguez-Granger J, Torres E, Puertas A, Navarro JM (2010). [Serological markers in immigrant and Spanish pregnant women in Granada]. Enferm Infecc Microbiol Clin.

[25] Valerio L, Barro S, Perez B, Roca C, Fernandez J, Solsona L (2008). Seroprevalence of chronic viral hepatitis markers in 791 recent immigrants in Catalonia, Spain. Screening and vaccination against hepatitis B recommendations. Rev Clin Esp.

[26] Lopez-Velez R, Huerga H, Turrientes MC (2003). Infectious diseases in immigrants from the perspective of a tropical medicine referral unit. Am J Trop Med Hyg.

[27] Gobbi F, Angheben A, Anselmi M, Postiglione C, Repetto E, Buonfrate D (2014). Profile of Trypanosoma cruzi infection in a tropical medicine reference center, Northern Italy. PLoS Negl Trop Dis.

[28] Munoz J, Gomez i Prat J, Gallego M, Gimeno F, Trevino B, Lopez-Chejade P (2009). Clinical profile of Trypanosoma cruzi infection in a non-endemic setting: immigration and Chagas disease in Barcelona (Spain). Acta Trop.

[29] Schar F, Trostdorf U, Giardina F, Khieu V, Muth S, Marti H (2013). Strongyloides stercoralis: Global Distribution and Risk Factors. PLoS Negl Trop Dis.

[30] Mascarello M, Gobbi F, Angheben A, Gobbo M, Gaiera G, Pegoraro M (2011). Prevalence of Strongyloides stercoralis infection among HIV-positive immigrants attending two Italian hospitals, from 2000 to 2009. Ann Trop Med Parasitol.

[31] Angheben A, Anselmi M, Andreoni F, Talamo M, Postiglione C, Gobbi F (2011). Strongyloidosis and Chagas disease sero-prevalence in a Bolivian community, Italy.

[32] Greenaway C, Dongier P, Boivin JF, Tapiero B, Miller M, Schwartzman K (2007). Susceptibility to measles, mumps, and rubella in newly arrived adult immigrants and refugees. Ann Intern Med.

[33] Andrus JK, de Quadros CA, Solorzano CC, Periago MR, Henderson DA (2011). Measles and rubella eradication in the Americas. Vaccine.

[34] van Rijckevorsel GG, Damen M, Sonder GJ, van der Loeff MF, van den Hoek A (2012). Seroprevalence of varicella-zoster virus and predictors for seronegativity in the Amsterdam adult population. BMC Inf Dis.

[35] Llenas-Garcia J, Rubio R, Hernando A, Arrazola P, Pulido F (2013). Do HIV-positive adult immigrants need to be screened for measles-mumps-rubella and varicella zoster virus immunization?. AIDS Care.

[36] Valerio L, Escriba JM, Fernandez-Vazquez J, Roca C, Milozzi J, Solsona L (2009). Biogeographical origin and varicella risk in the adult immigration population in Catalonia, Spain (2004–2006). Euro Surveill.

[37] Kempf W, Meylan P, Gerber S, Aebi C, Agosti R, Buchner S (2007). Swiss recommendations for the management of varicella zoster virus infections. Swiss Med Wkly.

