# Supplementary material for: Prevalence of chronic infections and susceptibility to measles and varicella-zoster virus in Latin American immigrants
Source: Infect Dis Poverty. 2016 May 11;5:41. doi: 10.1186/s40249-016-0136-7 (PMC4863343; doi:10.1186/s40249-016-0136-7)
Supplement: Additional file 1: — Multilingual abstracts in the five official working languages of the United Nations. (PDF 384 kb) [file 40249_2016_136_MOESM1_ESM.pdf]

Translation of the abstract into the five official working languages of the United Nations

انتشار الأمراض المزمنة والقابلية للإصابة بالحصبة بالفيروس النطاقي الحماقي لدى مهاجري أمريكا اللاتينية

ايفز جاكسون، ليليان سانتوس، إيزابيلا آرم فيرنيز، آن موريس، هانز وولف، فرانسوا شابفي، لوران جيتاز

الملخص

معلومات أساسية: وصلت أعداد كبيرة من المهاجرين من أمريكيا اللاتينية مؤخراً إلى أوروبا الغربية. ويأتي دور البرامج العلاجية والوقائية في مراعاة احتمالية أن يكونوا مصابين أو ناقلين للأمراض المزمنة الوافدة وقابليتهم للإصابة بالأمراض المرتحلة. وقد هدفنا إلى تقييم مدى انتشار ووجود الأمراض المزمنة الوافدة بين المهاجرين من الأمريكيين اللاتينيين، وقابليتهم للإصابة بالأمراض المرتحلة المنتشرة بشكل واسع.

المنهجية: تم إجراء تحاليل سيريولوجية على مشاركين متطوعين بالغين من المجتمع ومن مركز صحي أولي في جنيف 2008، لاختبار أمصال مخزنة لفيروس العوز المناعي البشري، الالتهاب الكبدي الفيروسي ب، الزهري، الاسطوانيات البرازية، المثقبية الكروزية، الحماق والحصبة. ولم نعتمد سوى الأمراض النشطة المزمنة في التحليل.

النتائج: خلصت النتائج إلى أن المشاركين الذين يبلغ عددهم 1012 مشارك، يبلغون من العمر 37.2 (المتوسط المعياري 11.3) سنة، كان أغلبهم من الإناث (82.5%) والبوليفيين (48%)، وإلى أن 209 (20.7%) منهم يحملون واحداً على الأقل من الأمراض المزمنة، بينما 27 (2.7%) منهم يحملون اثنان أو أكثر من الأمراض المزمنة. وكانت المثقبية الكروزية (12.8%) والاسطوانيات البرازية (8.4%) أكثر الأمراض النشطة المزمنة انتشاراً بالمقارنة مع الزهري (0.4%)، الالتهاب الكبدي الفيروسي ب (0.4%) وفيروز العوز المناعي (1.4%). كما وجد إصابة 28.2% و 18.5% من الحالات المصابة بالمثقبية الكروزية والاسطوانات البرازية بأمراض مصاحبة. وقد وجد ارتباط بين خطر التعدد المرضي والأصل البوليفي (نسبة الأرجحية المعدلة: 13.6؛ مجال موثوقية 95%: 3.2-57.9)، وبلغت قابلية الإصابة بالفيروس النطاقي الحماقي والحصبة نسبة 0.7% و 1.4% على التوالي. إن المهاجرين من أصل أمريكي لاتيني معرضون لخطر المضاعفات والاستنشاط المحتمل للأمراض الطفيلية المزمنة، إلا أن خطر إصابتهم بالأمراض النشطة الفيروسية والزهرية المزمنة ضئيل نسبياً.

الخلاصة: يلزم تحري وجود الأمراض الطفيلية المزمنة النشطة بشكل منهجي، خاصة بين البوليفيين. ولا يحتاج معدل الحماية العالية ضد الحصبة والفيروس النطاقي الحماقي إلى تدخلات وقائية خاصة.

Translated from English version into Arabic Maysa Orabi, through

TRANSLATORS
WITHOUT BORDERS

拉美移民的慢性感染情况及其对麻疹和水痘-带状疱疹病毒易感性的影响

Yves Jackson, Lilian Santos, Isabelle Arm-Vernez, Anne Mauris, Hans Wolff, François Chappuis, Laurent Getaz

摘要

引言：近来，大量拉美移民涌入西欧。制定防治规划时需要考虑这些移民罹患和传播输入性慢性感染的风险，以及他们对全球感染性疾病的易感性。本研究旨在评估拉美移民中输入性慢性感染的流行和共感染情况，以及他们对高度流行的全球感染性疾病的易感情况。

方法：2008年在日内瓦的社区和一家初级卫生中心招募成年受试者。应用储存的血清进行HIV、HBV、梅毒、粪类圆线虫、克氏锥虫、水痘-带状疱疹病毒和麻疹的血清学检查。本研究只分析慢性活动性感染。

结果：1 012位受试者的平均年龄为37.2岁（标准差：11.3岁），多数为女性（82.5%）和玻利维亚移民（48%）。209位受试者（20.7%）检测到1种或多种慢性活动性感染，多数为1种感染。27人（2.7%）感染2种或以上上述病原体。与梅毒（0.4%）、HBV（0.4%）和HIV（1.4%）相比，克氏锥虫（12.8%）和粪

类圆线虫（8.4％）感染较多。28.2%的克氏锥虫感染者和18.5％的粪类圆线虫感染者同时伴有其他感染。玻利维亚移民具有多重感染的风险（调整*OR*: 13.6; 95%置信区间: 3.2-57.9）。对水痘-带状疱症病毒和麻疹病毒的易感性分别为0.7%和1.4%。拉美移民中存在并发症和慢性寄生虫感染复发的风险。而慢性病毒感染和梅毒活动性感染的风险较低。
结论：有必要对拉美移民的慢性活动性寄生虫感染进行系统筛查，尤其是玻利维亚移民。但因对麻疹和水痘-带状疱症病毒的保护率高，不需要特殊的预防性干预措施。

Translated from English version into Chinese by Qian Menbao, edited by Yang Pin, through

CHINESE CENTER FOR DISEASE CONTROL AND PREVENTION
NATIONAL INSTITUTE OF PARASITIC DISEASES

Prévalence d'infections chroniques et sensibilité à la rougeole et au virus de la varicelle-zona chez des immigrés d'Amérique latine

Yves Jackson, Lilian Santos, Isabelle Arm-Vernez, Anne Mauris, Hans Wolff, François Chappuis, Laurent Getaz

Résumé
Contexte : un grand nombre d'immigrés d'Amérique latine sont récemment arrivés en Europe occidentale. Les programmes de traitement et de prévention doivent tenir compte de leur risque de contraction et de transmission d'infections chroniques importées ainsi que de leur sensibilité aux infections cosmopolites. Notre objectif a été d'évaluer la prévalence et la cooccurence d'infections chroniques importées parmi des immigrés d'Amérique latine ainsi que leur sensibilité à des infections cosmopolites à forte prévalence.
Méthodes : les participants adultes ont été recrutés parmi la communauté ainsi qu'au sein d'un centre de santé publique primaire à Genève en 2008. Des tests sérologiques ont été réalisés sur les sérums stockés afin de dépister la présence du VIH, du VHB, de la syphilis, du *Strongyloides stercoralis*, du *Trypanosoma cruzi*, de la varicelle et de la rougeole. Nous n'avons tenu compte que des infections chroniques actives dans notre analyse.
Résultats : les 1012 participants âgés en moyenne de 37,2 ans (ET 11,3) étaient majoritairement des femmes (82,5 %) et d'origine bolivienne (48 %). De manière générale, 209 personnes (20,7 %) présentaient au moins une infection chronique et 27 personnes (2,7 %) présentaient deux infections chroniques ou plus. Les infections à *T. cruzi* (12,8 %) et *S. stercoralis* (8,4 %) représentaient les infections chroniques actives les plus répandues par rapport à la syphilis (0,4 %), au VHB (0,4 %) et au VIH (1,4 %). Les infections concomitantes affectaient 28,2 % et 18,5 % des cas infectés par le *T. cruzi* et le *S. stercoralis* . L'origine bolivienne (*rapport de cotes ajusté*: 13,6 ; *IC* à 95 % : 3,2-57,9) était associée à un risque d'infections multiples. Les sensibilités au VZV et à la rougeole atteignaient respectivement 0,7 % et 1,4 %. Les immigrés d'Amérique latine courent un risque de complications et de réactivation possible d'infections parasitiques chroniques mais présentent de manière générale des risques plus faibles de contracter des infections chroniques virales et syphilitiques actives.
Conclusion : le dépistage systématique d'infections parasitiques chroniques actives est donc nécessaire, en particulier chez les Boliviens. Le degré élevé de protection contre la rougeole et le VZV ne requiert aucune intervention préventive.

Translated from English version into French by eric ragu, through

Распространение хронических инфекций и подверженность вирусам кори и ветряной оспы иммигрантов из Латинской Америки

Yves Jackson, Lilian Santos, Isabelle Arm-Vernez, Anne Mauris, Hans Wolff, François Chappuis, Laurent Getaz

Конспект

Обоснование: Большое количество иммигрантов из Латинской Америки недавно прибыло в Западную Европу. Программы профилактических и лечебных мер должны учитывать риск носительства и передачи хронических заболеваний и подверженности иностранным инфекциям. Наша цель – оценить распространение и возникновение ввозимых хронических инфекций у иммигрантов из Латинской Америки, их восприимчивость к всемирно широко распространённым инфекциям.

Методы: Взрослые участники набирались по месту жительства и в главном здравоохранительном центре в Женеве в 2008 году. Производились анализы серологической реакции с использованием сохраненной сыворотки крови на HIV, HBV, сифилис, *Strongyloides stercoralis*, *Trypanosoma cruzi*, ветряную оспу и корь. В этом исследовании мы рассматривали только хронические активные инфекции.

Результаты: 1 012 участника, в возрасте 37,2 (SD 11.3) года, в основном женщины (82,5%), боливийцы (48%). В целом, 209 (20,7%) имеют хотя бы одну и 27 (2,7%) две или более, хроническую инфекцию. *T. cruzi* (12,8%) и *S. stercoralis* (8,4%) были самыми распространенными хроническими активными инфекциями, в то время, как сифилис – (0,4%), HBV (0.4%) и HIV (1.4%). Сопутствующие инфекции выявлены в 28,2% и 18,5% случаях инфицирования *T. cruzi* и *S. stercoralis*. Происхождение из Боливии (a*OR*: 13.6; 95%*CI*: 3.2-57.9) оказалось связано с риском множественных инфекций. Подверженность ветряной оспе и кори была 0,7% и 1,4%, соответственно. Иммигранты из Латинской Америки вносят риск усложнения и возможной реактивации хронических паразитарных инфекций, но в целом несут малый риск хронических вирусных инфекций и сифилиса.

Заключение: Систематическая проверка на хронические активные паразитарные инфекции необходима, особенно среди боливийцев. Высокая степень защищенности от ветряной оспы и кори не требует особых профилактических мероприятий.

Translated from English version into Russian by Anna Philippova, through

Prevalencia de las infecciones crónicas y susceptibilidad al sarampión y el virus de la varicela-zóster en inmigrantes de Latinoamérica

Yves Jackson, Lilian Santos, Isabelle Arm-Vernez, Anne Mauris, Hans Wolff, François Chappuis, Laurent Getaz

Resumen

Antecedentes: Recientemente ha llegado una gran cantidad de inmigrantes de Latinoamérica a Europa Occidental. Los programas curativos y preventivos tienen que tener en cuenta el riesgo de que padezcan o transmitan infecciones crónicas importadas y de su susceptibilidad a las infecciones cosmopolitas. Nuestro objetivo fue evaluar la prevalencia y la co-ocurrencia de las infecciones crónicas importadas entre los inmigrantes latinoamericanos, y su susceptibilidad a infecciones cosmopolitas de alta prevalencia.

Métodos: Se reclutaron participantes adultos en la comunidad y en un centro de atención médica primaria en Ginebra en el año 2008. En el suero almacenado se llevaron a cabo pruebas serológicas para VIH, VHB, sífilis, *Strongyloides stercoralis*, *Trypanosoma cruzi*, varicela y sarampión. En el análisis solo consideramos las infecciones crónicas activas.

Resultados: Los 1012 participantes, de 37,2 de edad (SD 11,3) fueron principalmente mujeres (82,5%) y de Bolivia (48%). En general, 209 (20,7%) padecían de por lo menos una y 27 (2,7%) de dos o más infecciones crónicas. *T. cruzi* (12,8%) y *S. stercoralis* (8,4%) fueron las infecciones crónicas activas con mayor prevalencia en comparación con sífilis (0,4%), VHB (0,4%) y VIH (1,4%). Las infecciones concomitantes afectaban a 28,2% y 18,5% de los casos infectados por *T. cruzi* y *S. stercoralis.* Bolivia como lugar de origen (a *OR*: 13,6; 95%*IC*: 3,2-57,9) estaba asociado a riesgo de múltiples infecciones. La susceptibilidad para el virus de la varicela-zóster y del sarampión fue de 0,7% y 1,4% respectivamente. Los inmigrantes de Latinoamérica corren riesgo de complicaciones y una posible reactivación de infecciones crónicas parasitarias pero en general tienen menor riesgo de infecciones virales crónicas y de infecciones activas por sífilis.

Conclusión: Se necesitan de sistemas de diagnóstico sistemáticos para infecciones parasitarias crónicas activas en particular entre la población de Bolivia. El elevado índice de protección contra el sarampión y el virus de la varicela-zóster no requiere de intervenciones preventivas específicas.

Translated from English version into Spanish by Maria Alejandra Aguada, through

TRANSLATORS
WITHOUT BORDERS
